# Effects of polyacrylamide molecular weight and mass concentration on water transport characteristics of iron tailings

**DOI:** 10.1038/s41598-021-85338-8

**Published:** 2021-03-18

**Authors:** Bo Sun, Chunjuan Lyu, Rutian Bi, Lu Xia, Xu Zhang, Caicai Xu, Yu Wang, Yansong Guo

**Affiliations:** 1 College of Resources and Environment, Shanxi Agricultural University, Taigu, 030801 Shanxi China; 2 College of Resources and Environment, National Experimental Teaching Demonstration Center, Shanxi Agricultural University, Taigu, 030801 Shanxi China

**Keywords:** Environmental sciences, Environmental chemistry, Materials chemistry

## Abstract

Iron tailings have few macropores which severely inhibit infiltration and transport of soil water. Polyacrylamide (PAM) can regulate soil water, but it is rarely used when remediating tailings matrix. In this research, PAM of four molecular weights of 300w, 600w, 800w, and 1000w were selected as amendments, and were each applied at five mass concentrations of 0% (CK), 0.01%, 0.04%, 0.08%, and 0.16% to observe their effects on water transport in iron tailings using column simulations in the laboratory. After adding PAM, the water retention and saturated water content of iron tailings increased significantly (*P* < 0.05). With increases in PAM molecular weight and mass concentration, the saturated hydraulic conductivity showed a downward trend, but the saturated hydraulic conductivity increased after a dry–wet cycle. With the increase of PAM mass concentration, adding PAM of 1000w molecular weight to iron tailing decreased infiltration capacity, but treatments of other molecular weights all initially increased then decreased infiltration capacity. The greatest improvement on infiltration capacity of iron tailings was observed with the addition of PAM of 300w molecular weight and 0.01% mass concentration. Adding PAM increased the vertical depth of the saturation zone of iron tailings (*P* < 0.05) with a maximum depth of 20.83 cm. The Kostiakov model more accurately simulated water infiltration of iron tailings compared with the Horton and Philip models. On the whole, when PAM of low molecular weight and concentration was added to iron tailings, PAM increased stable infiltration, saturated water content, and water retention. It also inhibited saturated hydraulic conductivity of iron tailings. Therefore, in practice, it is necessary to select the appropriate molecular weight and mass concentration of PAM according to the dominant limiting factors and remediation needs of the matrix.

## Introduction

Tailings are the wastes from mineral separation. There is a large amount of iron tailings in China but its utilization rate is far lower than developed countries. Due to the fine particles and loose structure of iron tailings, dust or run off is easily produced under rainfall, which threatens the surrounding ecological environments^1–3^. A large number of iron tailings are open air dumps, which not only occupy land and pollute the environment, but also may collapse and seriously endanger the safety of people’s lives and property. Therefore, the artificial reclamation of abandoned tailings is not only needed to improve waste management, but also to protect the ecological environment and ensure safety. Generally speaking, covering the surface of tailings with soil is an effective reclamation. However, most tailings are dumped in mountainous areas in China, where rocks are abundant but soil is scare, or in some places, not available. Covering with soil is also costly and time-consuming. Therefore, it is necessary to add amendments to improve the physical and chemical properties for iron tailings^4^. PAM is a kind of synthetic polymer, which is either anionic, cationic, or non-ionic depending on its structure. Anionic PAM is widely used in cropland water retention, soil erosion control, and other soil management strategies^5–8^ because of its low cost and stability, and because it is non-toxic and harmless to the environment.

Soil water status is an important factor affecting vegetation restoration, and soil water transport is affected by soil texture, bulk density, and pore distribution. Under natural conditions, iron tailings water transport capacity is lower than that of cropland soil^9^, and the air infiltration is poor, which seriously inhibits plant root growth. Anionic PAM can regulate soil water infiltration, enhance soil water retention capacity, and inhibit soil water evaporation. Flanagan et al.^10^ added a small amount of anionic PAM to tap water under artificial rainfall, and the results showed that the addition of PAM greatly increased iron tailings water infiltration. Tümsava et al.^11^ compared the effects of different PAM mass concentrations on slope runoff and water infiltration after artificial rainfall, and the results showed that the most effective PAM mass concentrations were 3.333 kg·ha^−1^ and 5.000 kg·ha^−1^ for reducing soil erosion and increasing infiltration, respectively. Soil crusting will result in a significant reduction in soil water infiltration, and the application of PAM can effectively alleviate the production of crusts^12^. Zhang et al.^13^ sprayed PAM on the surface of loam, and further confirmed that PAM inhibited the formation of soil surface crusts to increased soil water infiltration. The effect of PAM application is influenced by soil texture, PAM molecular weight, PAM mass concentration, and method of application^14^. Yu et al.^15^ compared the effect of PAM with different molecular weights on loam and found that molecular weights of 150w and 180w PAM increased soil infiltration significantly more than 120w, and 150w PAM was slightly better than 180w. Yuan et al.^16^ found that PAM significantly increased soil water infiltration at 0.01% mass concentration, and it inhibits soil infiltration if PAM mass concentration exceeds 0.04%; when the mass concentration of PAM was 0.16%, the soil was almost impermeable to water. Therefore, the PAM mass concentration has a certain effect on the water infiltration in soil.

The addition of PAM and other soil amendments also stabilizes soil structure, maintains air infiltration of soil, and increases soil saturated hydraulic conductivity^17^. Fei et al.^18^ added PAM and biochar to saline soil and reported a significant increase in saturated hydraulic conductivity, and the effect was greatest at 5% biochar and 1% PAM. Pan et al.^19^ applied PAM and phosphogypsum to silt loam soil and showed that PAM applied to the soil surface significantly increased soil saturated hydraulic conductivity, and adding phosphogypsum further promoted this effect.

Past research on the application of PAM has been mostly focused on water and nutrient retention in cropland, and controlling soil erosion on sloping cropland^20–22^. But research based on mining area reclamation is relatively rare, especially for the application of PAM in water transport of iron tailings. In this research, PAM were applied to iron tailings for the first time to explore the effects of molecular weights and mass concentrations of PAM on the water transport and water retention of iron tailings. The main purposes of this study were (1) to confirm whether PAM can improve the saturated hydraulic conductivity of iron tailings, (2) to characterize the water infiltration of PAM treatments in iron tailings and to identify the fitting model in water infiltration, (3) to compare the improvement effect of different PAM molecular weights on water retention of iron tailings, and (4) to propose the PAM application recommendation in iron tailings. The research results will be helpful for the ecological restoration of similar mining areas, especially for the reclamation of soilless mining area.

## Results

### Saturated hydraulic conductivity

#### Saturated water content

**Figure 1:**
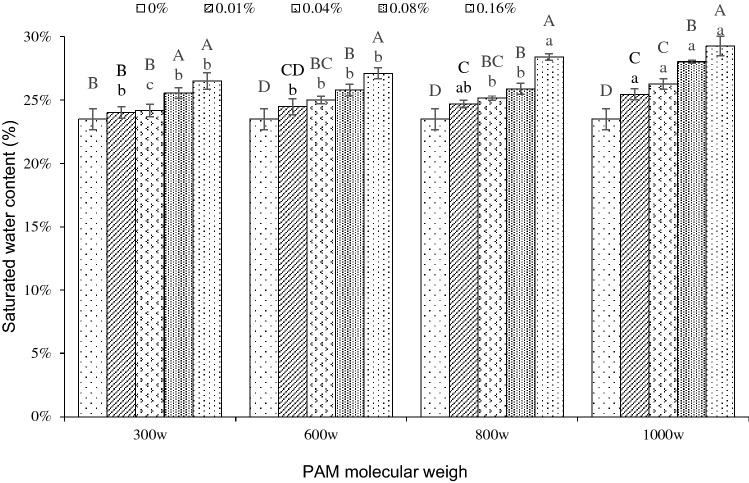
Saturated water content of iron tailings for four molecular weight (300w, 600w, 800w and 1000w) and five mass concentration (0%, 0.01%, 0.04%, 0.08% and 0.16%) of PAM. Different capital letters indicate significant differences in saturated water content under different mass concentrations of PAM with the same molecular weight, and different lowercase letters indicate a significant difference in saturated water content under different molecular.

The effect of different treatments on the saturated water content of iron tailings is shown in Fig. 1. The saturated water content of iron tailings with all four molecular weights of PAM added all showed an upward trend with the increase of PAM mass concentration. When tailings had PAM added at mass concentrations of 0.01% and 0.04% with a molecular weight of 300w, there were no significant differences in saturated water content compared with CK. However, the saturated water content of the other treatments was significantly higher than CK (*P* < 0.05). As the molecular weight increased, the effect of PAM increased significantly. When the molecular weight was 300w, the saturated water content of the iron tailings with PAM mass concentrations of 0.01%, 0.04%, 0.08%, and 0.16% increased by 2.22%, 2.89%, 8.79%, and 12.88% compared to CK, respectively. When the PAM molecular weight was 1000w, the saturated water content of the iron tailings with PAM mass concentrations of 0.01%, 0.04%, 0.08%, and 0.16% increased by 8.41%, 11.85%, 19.34%, and 24.55% compared with CK, respectively. The results show that PAM can improve the saturated water content of iron tailings.

The saturated water content of the iron tailings also increased as the molecular weight increased within the groups of the same PAM mass concentrations, but when the mass concentration of PAM was low, the increase in molecular weight had no effect on the change of saturated water content of the iron tailings. When the mass concentration of PAM was 0.01%, the saturated water content of the iron tailings treated with molecular weights of 300w, 600w, 800w, and 1000w were 24.01%, 24.49%, 24.70%, and 25.46%, respectively, which were only 2.22%, 4.27%, 5.16%, and 8.41% higher than CK, respectively. When the PAM mass concentration was 0.16%, the saturated water content of the iron tailings with the molecular weights of 300w, 600w, 800w, and 1000w increased by 12.88%, 15.40%, 20.96%, and 24.55% compared with CK, respectively. When the mass concentrations of PAM were higher, there was a greater increase in saturated water content with increasing molecular weights compared with PAM with low mass concentration.

#### Saturated hydraulic conductivity

**Figure 2:**
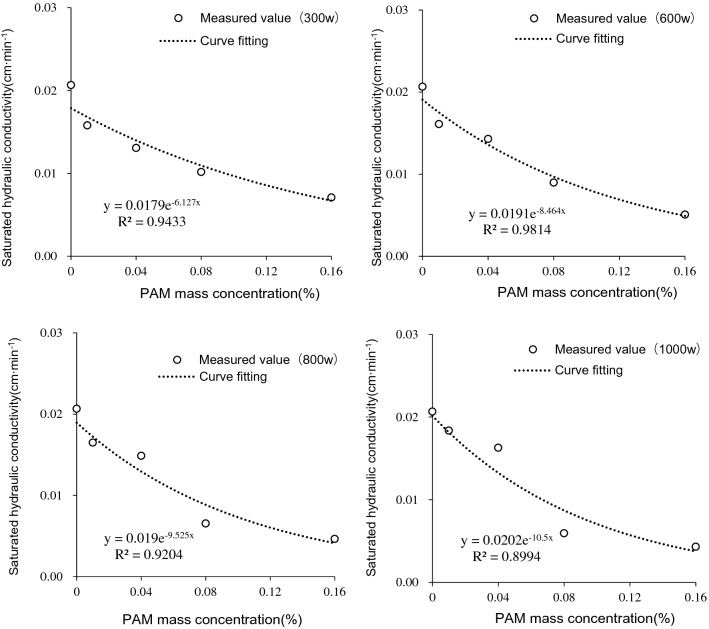
Saturated hydraulic conductivity of iron tailings for four molecular weight (300w, 600w, 800w and 1000w) and five mass concentration (0%, 0.01%, 0.04%, 0.08% and 0.16%) of PAM.

The effect of PAM with different molecular weights and mass concentrations on the saturated hydraulic conductivity of iron tailings is shown in Fig. 2. When the molecular weight of PAM was constant, the saturated hydraulic conductivity of iron tailings gradually decreased with increased PAM mass concentrations. An exponential function equation best described the relationship between saturated hydraulic conductivity of iron tailings and PAM mass concentration, with a R^2^ value between 0.8994 and 0.9814.

Comparing the saturated hydraulic conductivity of treatments with different molecular weights under the same PAM mass concentration, it was found that the effect of molecular weight on saturated hydraulic conductivity differed between high PAM mass concentrations (0.08% and 0.16%) and low PAM mass concentrations (0.01% and 0.04%). When the mass concentration of PAM was 0.01% and 0.04%, the saturated hydraulic conductivity of iron tailings both tended to increase with an increase in PAM molecular weight. However, when the mass concentrations of PAM were 0.08% and 0.16%, the saturated hydraulic conductivity of iron tailings decreased with increased PAM molecular weight. This shows that when the PAM mass concentration is low, increasing the molecular weight of PAM promotes greater rates of saturated hydraulic conductivity of iron tailings.

**Figure 3:**
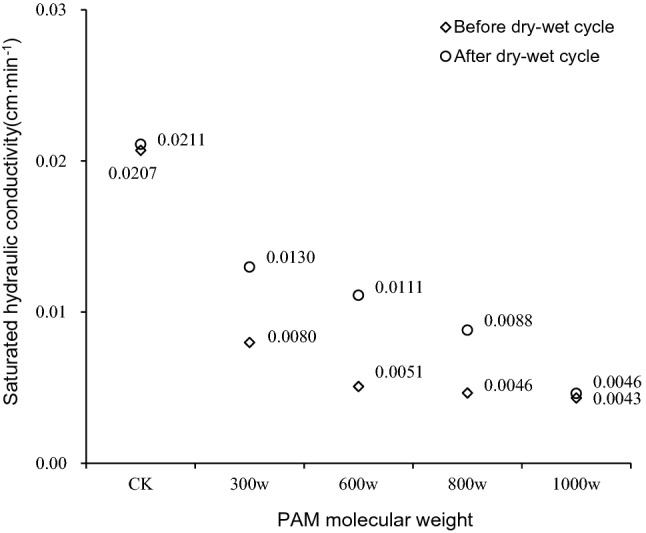
Saturated hydraulic conductivity of iron tailings under a dry–wet cycle for four molecular weight (300w, 600w, 800w and 1000w) and five mass concentration (0%, 0.01%, 0.04%, 0.08% and 0.16%) of PAM.

To study the effect of dry–wet cycles on saturated hydraulic conductivity of iron tailing with PAM added, the soil column with the mass concentration of 0.16% from the water retention experiment was re-saturated after air drying. The saturated hydraulic conductivity of the iron tailings before and after a dry–wet cycle is shown in Fig. 3. When the molecular weights were 300w, 600w, and 800w, the saturated hydraulic conductivity significantly increased by 62.44%, 118.76%, and 89.44% compared to before the dry–wet cycle, respectively. However, for the CK and the 1000w molecular weight of PAM treatment, there were no significant increases in saturated hydraulic conductivity when compared to before the dry–wet cycle as those treatments only increased by 1.93% and 7.04%, respectively. The results show that the dry–wet cycle weakens PAMs inhibition of saturated hydraulic conductivity of tailings, but how wet-dry cycles change saturated hydraulic conductivity of tailings at higher molecular weights needs to be further explored.

### Water infiltration characteristics

#### Water infiltration rate

The effect of PAM molecular weight and mass concentration on the infiltration characteristic values of iron tailings is shown in Table 1. The initial infiltration rate, average infiltration rate, and steady infiltration rate are usually used as three indicators to characterize soil water infiltration capacity. When the molecular weight was constant, the average infiltration rate of all treatments decreased with an increase of mass concentration compared with CK.

**Table 1:** Initial infiltration rate, average infiltration rate and stable infiltration rate for four molecular weight (300w, 600w, 800w and 1000w) and five mass concentration (0%, 0.01%, 0.04%, 0.08% and 0.16%) of PAM.

PAM molecular weight	PAM mass concentration (%)	Initial infiltration rate ± SD (cm min^−1^)	Average infiltration rate ± SD (cm min^−1^)	Stable infiltration rate ± SD (cm min^−1^)
300w	0	0.567 ± 0.034 BC	0.242 ± 0.002 A	0.034 ± 0.002 E
	0.01	0.489 ± 0.019 Da	0.174 ± 0.011 Bb	0.072 ± 0.002 Aa
	0.04	0.633 ± 0.034 Ab	0.161 ± 0.007 Cb	0.068 ± 0.001 Ba
	0.08	0.589 ± 0.019 Abb	0.133 ± 0.002 Dc	0.053 ± 0.001 Ca
	0.16	0.533 ± 0.001 CDb	0.099 ± 0.003 Ea	0.039 ± 0.003 Da
600w	0	0.567 ± 0.034 C	0.242 ± 0.002 A	0.034 ± 0.002 B
	0.01	0.584 ± 0.020 Ca	0.193 ± 0.003 Ba	0.046 ± 0.002 Ac
	0.04	0.712 ± 0.009 Aa	0.190 ± 0.002 Ba	0.044 ± 0.001 Ab
	0.08	0.679 ± 0.003 Aba	0.158 ± 0.004 Cb	0.034 ± 0.001 Bb
	0.16	0.656 ± 0.020 Ba	0.093 ± 0.011 Da	0.021 ± 0.001 Cb
800w	0	0.567 ± 0.034 A	0.242 ± 0.002 A	0.034 ± 0.002 B
	0.01	0.555 ± 0.069 ABa	0.186 ± 0.006 Bab	0.053 ± 0.005 Ab
	0.04	0.518 ± 0.015 ABCc	0.183 ± 0.003 Ba	0.034 ± 0.002 Bc
	0.08	0.491 ± 0.005 BCc	0.181 ± 0.004 Ba	0.029 ± 0.001 Bc
	0.16	0.456 ± 0.020 Cc	0.093 ± 0.007 Ca	0.019 ± 0.001 Cb
1000w	0	0.567 ± 0.034 A	0.242 ± 0.002 A	0.034 ± 0.002 A
	0.01	0.533 ± 0.001 Aa	0.150 ± 0.002 Bc	0.028 ± 0.001 Bd
	0.04	0.478 ± 0.019 Bd	0.144 ± 0.001 Cc	0.025 ± 0.001 Cd
	0.08	0.449 ± 0.016 Bd	0.119 ± 0.003 Dd	0.021 ± 0.001 Dd
	0.16	0.411 ± 0.019 Cd	0.091 ± 0.004 Ea	0.014 ± 0.002 Ec

S.D. means the standard derivation among the repetitions (n = 3). Different capital letters indicate the significant difference in infiltration rate under different mass concentrations of PAM with the same molecular weight, and different lowercase letters indicate the significant difference in infiltration rate under different molecular weight PAM treatments when the mass concentration of PAM is the same.

The initial infiltration rate and stable infiltration rate presented different variations with PAM molecular weight and mass concentration. When PAM molecular weight was 300w and 600w, the initial infiltration rate and stable infiltration rate both showed a trend of increasing first followed by a decrease. With the 300w and 600w PAM molecular weight treatments, the maximum initial infiltration rates were 0.663 and 0.712 cm·min^−1^, respectively, when the mass concentration was 0.04%, and the maximum initial infiltration rates in both treatments were significantly greater than the maximum initial infiltration rate in the CK treatment, which averaged 0.567 cm min^−1^. In the 300w and 600w PAM molecular weight treatments, the maximum stable infiltration rates were 0.072 and 0.046 cm min^−1^, respectively, at a mass concentration of 0.01%, and both treatments were significantly higher than CK which averaged 0.034 cm min^−1^. When the molecular weight of PAM was 800w and 1000w, the initial infiltration rates and stable infiltration rates of iron tailings showed a gradual decrease with increasing mass concentrations.

When the molecular weight and mass concentrations of PAM are low, it effectively increases the initial infiltration rate and stable infiltration rate of the iron tailings, and high molecular weight PAM at various concentrations reduces the initial infiltration rate and stable infiltration rate of tailings.

#### Wet peak distance

**Figure 4:**
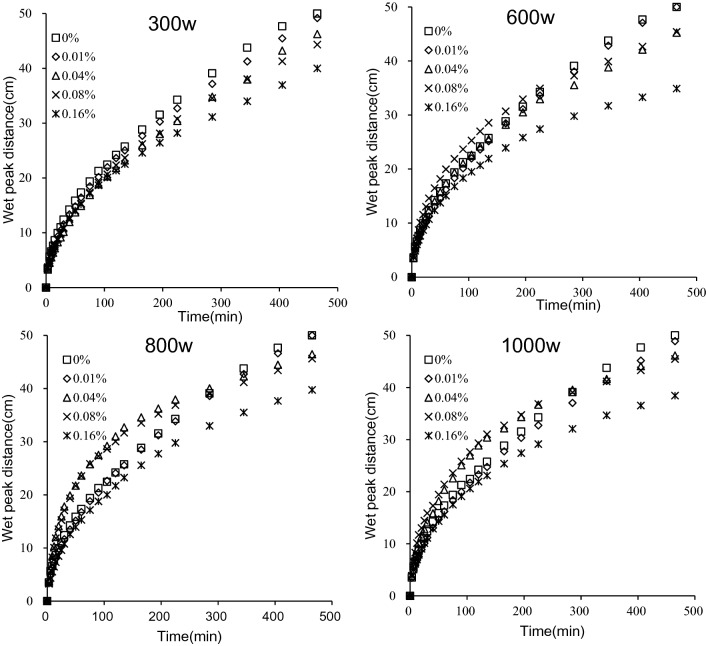
Wet peak distance of iron tailings for four molecular weight (300w, 600w, 800w and 1000w) and five mass concentration (0%, 0.01%, 0.04%, 0.08% and 0.16%) of PAM.

The effect of PAM molecular weight and mass concentration on the wet peak distance of iron tailings is shown in Fig. 4. In the wet peak, the soil water gradient is large, so there will be a large soil water force to drive the wet peak movement downward. The wet peak moved fast in thee 100 min before the start of the experiment, then tended to be flat. Under the same PAM molecular weight treatment, the PAM mass concentration had a significant effect on the wet peak distance (*P* < 0.05). At the end of the infiltration experiment, the wet peak distance of iron tailings under the four molecular weight treatments all decreased with increasing mass concentration.

However, during infiltration, the wet peak distance of iron tailings with different PAM molecular weight treatments differed with increasing PAM mass concentrations. When the PAM of 300w molecular weight was added to tailings, the wet peak distance increased with the increase of PAM mass concentrations. When PAM of molecular weights 600w, 800w, and 1000w were added to tailings, the relative size of the wetting front distance at infiltration lasted about 300 min. In the initial 300 min of infiltration, the wetting front distance of iron tailings under different PAM concentrations was larger than that of CK, but after 300 min, it gradually lagged behind CK. This phenomenon indicated that PAM added to iron tailings may be partially dissolved during the initial stage of infiltration, which changes the pore structure of tailings and increases the connectivity of pore water, and promotes the transport of wetting front. As the infiltration time increased, a saturated zone appeared in the wetting front. PAM dissolved fully and blocked part of the pores in the tailings thereby decreasing the speed of the wetting front, but the rate of the movement of the wetting front was still greater than that of CK.

#### Cumulative infiltration volume

**Figure 5:**
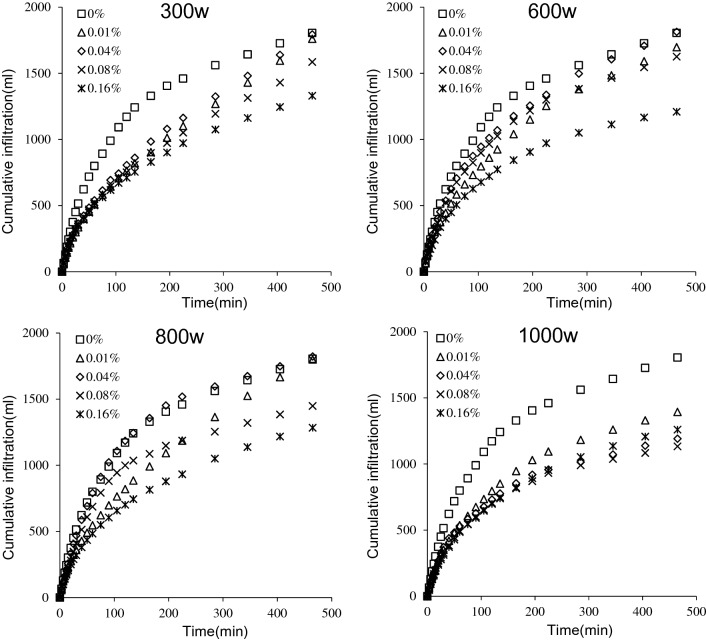
Cumulative infiltration of iron tailings for four molecular weight (300w, 600w, 800w and 1000w) and five mass concentration (0%, 0.01%, 0.04%, 0.08% and 0.16%) of PAM.

The cumulative infiltration volume can be calculated by the drop in height of level of liquid in the Mariotte bottle. It was seen that changes in cumulative infiltration volume of different treatments followed patterns similar to that of the wetting front in Fig. 5. In the first 100 min of infiltration, the infiltration volume increased at a faster rate, and then gradually stabilized. As the molecular weight of PAM increased, the cumulative infiltration volume showed different trends than were observed with increases in mass concentration. When the PAM of molecular weight 300w was added to tailings, the cumulative infiltration volume according to the mass concentration from largest to smallest were 1404.16 ml, 1013.04 ml, 1079.72 ml, 973.29 ml, and 901.48 ml in the first 100 min, which was decreased by 27.64%, 22.89%, 30.47%, and 35.59% compared with CK. When PAM with molecular weights 600w and 800w were added to tailings, the cumulative infiltration volume at PAM mass concentrations of 0.04% were 0.48% and 1.02% higher than CK, respectively at the end of the infiltration. When the PAM of molecular weight 1000w was added to tailings, the cumulative infiltration volume was significantly lower than CK (*P* < 0.05). Because PAM absorbed and retained water, adding it will affect the speed of water transport in soil to a certain extent. When low molecular weight PAM was added to soil, the inhibition of water transport in iron tailings was not observed. As the molecular weights increased, PAM-induced inhibition of water transport was observed. The experiments showed that PAM increased the cumulative infiltration volume when the molecular weight of PAM was 600w and 800w and the mass concentration was between 0.04 and 0.08%.

#### Water content of the tailings profile

**Figure 6:**
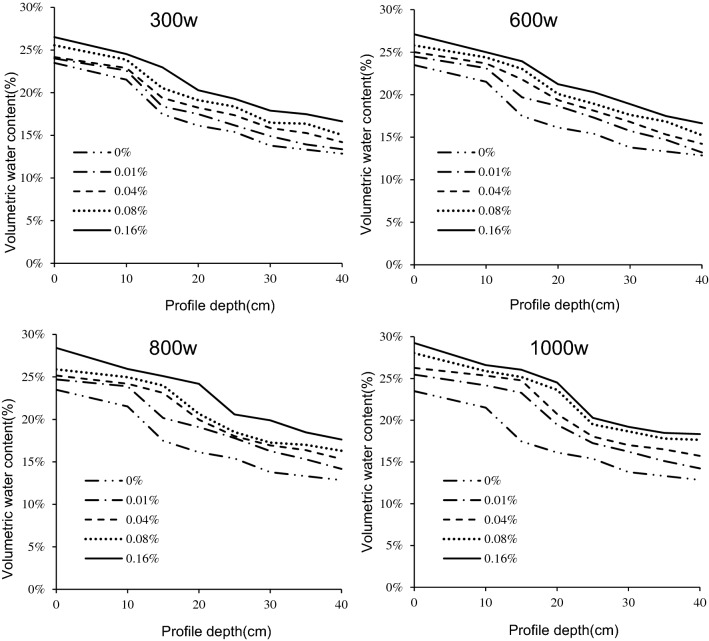
Volumetric water content of iron tailings profile for four molecular weight (300w, 600w, 800w and 1000w) and five mass concentration (0%, 0.01%, 0.04%, 0.08% and 0.16%) of PAM.

There was water stratification between the iron tailings aquifers (Fig. 6). The water content decreased and the stratification weakened with tailings depth. According to the profile water distribution gradient, the iron tailings profile was divided into 4 zones from top to bottom: saturation zone, transition zone, transmission zone, and wet zone^8^. After the end of the experiment, the soil column for each treatment had a clear boundary between the saturated and unsaturated zones, and the depth of this delineation was between 13 and 22 cm (Table 2). The water content of iron tailings at the top of the saturated zone was usually above 20%, and was significantly lower in the unsaturated zone. This saturated boundary depth gradually deepened with an increase in the molecular weight and mass concentration of PAM. This indicates that the addition of PAM can increase the depth of the water saturation zone of iron tailings, and its effect increased with the increased molecular weight of PAM.

**Table 2:** Saturated boundary depth of soil water stratification for four molecular weight (300w, 600w, 800w and 1000w) and five mass concentration (0%, 0.01%, 0.04%, 0.08% and 0.16%) of PAM.

PAM molecular weight	PAM mass concentration (%)	Boundary depth ± SD (cm)
300w	0	13.57 ± 0.35D
	0.01	13.63 ± 0.25CDc
	0.04	14.00 ± 0.10BCd
	0.08	14.40 ± 0.10Bd
	0.16	15.90 ± 0.20Ac
600w	0	13.57 ± 0.35E
	0.01	14.37 ± 0.25Db
	0.04	15.60 ± 0.26Cc
	0.08	16.43 ± 0.32Bc
	0.16	17.60 ± 0.36Ab
800w	0	13.57 ± 0.35E
	0.01	14.83 ± 0.31Db
	0.04	16.23 ± 0.42Cb
	0.08	18.17 ± 0.31Bb
	0.16	20.83 ± 0.31Aa
1000w	0	13.57 ± 0.35D
	0.01	15.83 ± 0.46Ca
	0.04	18.77 ± 0.35Ba
	0.08	20.57 ± 0.32Aa
	0.16	20.97 ± 0.83Aa

S.D. means the standard derivation among the repetitions (n = 3). Different capital letters indicate the significant difference in saturated boundary depth of soil water under different mass concentrations of PAM with the same molecular weight of PAM, and different lowercase letters indicate the significant difference in saturated boundary depth of soil water under different molecular weight PAM treatments when the mass concentration of PAM is the same.

The water content of each iron tailings layer all increased compared to CK after adding PAM (Fig. 6). When PAM with molecular weight of 300w was added to iron tailings, the difference of water content among PAM mass concentrations at the same depth was not significant (*P* < 0.05). Adding PAM with molecular weights of 600w, 800w, and 1000w to iron tailings, when the molecular weight was the same, the water content among PAM mass concentrations at the same depth was significantly different. Multiple comparisons showed that the differences were mainly between the 0 and 0.01% treatments, and the differences were not significant among other PAM mass concentrations treatments.

The water content of tailings at the same depth increased with increased molecular weight and mass concentration of PAM, and the difference between different depths gradually decreased. This indicated that the application of PAM to iron tailings increased water retention. When the molecular weight and mass concentration of PAM increased, the depth of the water saturation zone of iron tailings tended to gradually deepen, and the difference in water content among depths gradually decreased.

#### Model fitting for water infiltration

Table 3 shows the fitting results for the Philip, Horton, and Kostiakov models for iron tailings treated by PAM with different molecular weights and mass concentrations. In the Philip model, S is the soil infiltration rate, which represents soil infiltration capacity. The larger the S value, the stronger the soil infiltration capacity. The change in the modelled S value was not consistent with the actual measurement, and there were several negative values in the stable infiltration rate represented by parameter A in the Philip model, indicating that some of the simulated values were smaller than the actual measured values. In the Horton model, the infiltration coefficient k was the difference between the initial and stable infiltration rates, and this approximately conformed to the changes observed in the measured infiltration rate. In this model, the c value represents the stable infiltration rate, and it was found to be larger than the actual measured value.

**Table 3:** Fitting of water infiltration process of iron tailings for four molecular weight (300w, 600w, 800w and 1000w) and five mass concentration (0%, 0.01%, 0.04%, 0.08% and 0.16%) of PAM.

PAM molecular weigh	PAM mass concentration (%)	Horton model					Philip model				Kostiakov model			
		α	k	c	R^2^	RMSE	S	A	R^2^	RMSE	a	b	R^2^	RMSE
ck	0	0.0581	0.5958	0.0196	0.9858	0.0213	2.5559	0.0295	0.9159	0.0726	1.1120	0.4034	0.8821	0.0646
300w	0.01	0.0902	0.5251	0.0501	0.9775	0.0177	1.7941	0.0252	0.9752	0.0185	0.8410	0.4285	0.9816	0.0160
	0.04	0.1029	0.7549	0.0853	0.9424	0.0347	2.0018	0.0024	0.9748	0.0229	1.1197	0.5038	0.9750	0.0229
	0.08	0.0852	0.6177	0.0610	0.9358	0.0348	2.0852	0.0013	0.9809	0.0187	1.0372	0.4955	0.9810	0.0187
	0.16	0.0666	0.5741	0.0499	0.9641	0.0263	2.0871	-0.0078	0.9654	0.0252	1.0159	0.5013	0.9653	0.0257
600w	0.01	0.0932	0.6369	0.0539	0.9503	0.0325	2.1798	0.0116	0.9427	0.0348	1.0236	0.4552	0.9494	0.0331
	0.04	0.0864	0.6523	0.0376	0.9350	0.0437	2.6204	0.0011	0.9801	0.0241	1.2469	0.4768	0.9834	0.0229
	0.08	0.0780	0.6120	0.0359	0.9150	0.0482	2.5213	-0.0034	0.9657	0.0306	1.1989	0.4824	0.9685	0.0302
	0.16	0.0713	0.5614	0.0568	0.8042	0.0620	1.9373	0.0085	0.9402	0.0312	0.9571	0.5100	0.9394	0.0316
800w	0.01	0.0988	0.6197	0.0611	0.9449	0.0313	2.0294	0.0169	0.9726	0.0221	0.9733	0.4552	0.9747	0.0212
	0.04	0.0320	0.4974	0.0129	0.9938	0.0123	2.1653	0.0513	0.8009	0.0701	0.9358	0.3668	0.8668	0.0592
	0.08	0.0198	0.4383	0.0137	0.9728	0.0235	2.0221	0.0227	0.8188	0.0613	0.8721	0.4007	0.8624	0.0552
	0.16	0.0619	0.4856	0.0415	0.9663	0.0224	1.8848	-0.0038	0.9614	0.0241	0.8828	0.4784	0.9659	0.0237
1000w	0.01	0.0749	0.5398	0.0451	0.9314	0.0350	2.0399	0.0013	0.9705	0.0229	0.9664	0.4741	0.9746	0.0220
	0.04	0.0384	0.4433	0.0240	0.9207	0.0382	1.9047	0.0044	0.8251	0.0567	0.8473	0.4413	0.8447	0.0546
	0.08	0.0475	0.4355	0.0301	0.9684	0.0216	1.8491	-0.0029	0.9481	0.0277	0.8488	0.4681	0.9567	0.0268
	0.16	0.0547	0.4111	0.0298	0.9625	0.0218	1.733	0.0034	0.9337	0.0295	0.7820	0.4488	0.9491	0.0272

α, k, a and b, infiltration experience parameters; S, water absorption rate; A and c, stable infiltration rate; R^2^, coefficient of determination; RMSE, root mean squared error.

In the Kostiakov model, a parameter is the infiltration coefficient, and the b parameter represents the degree of decrease in the infiltration rate over time. The change of parameters a and b under each treatment were generally consistent with the changes in the initial infiltration rate with the associated PAM molecular weight and mass concentration treatments. In this model, the simulated values had smaller errors, which shows that this model better reflects the changes in infiltration rates during infiltration.

The coefficient of determination R^2^ values for the Philip model fit was between 0.8009 and 0.9809, the mean value was 0.9336, and the root mean square error (RMSE) value was 0.0349. The coefficient of determination R^2^ values for the Horton model was between 0.8042 and 0.9938, the mean value was 0.9453, the RMSE was 0.0310. The coefficient of determination R^2^ values for the Kostiakov model was between 0.8447 and 0.9834, the mean value was 0.9424, and the RMSE was 0.0327. In terms of fitting accuracy, the Horton model had the highest accuracy, followed by the Kostiakov model, and the Philip model had the worst. However, the Kostiakov model was the best fit model based on the comprehensive fitting effect of the three models.

**Figure 7:**
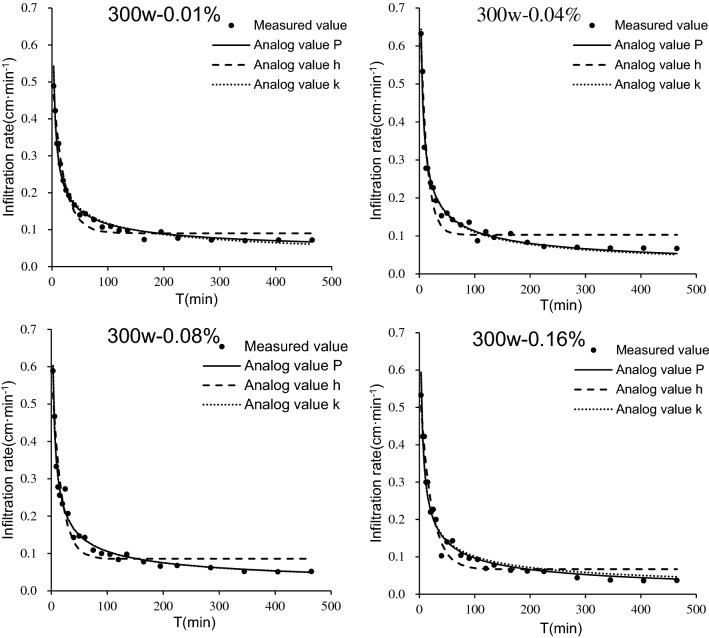
Infiltration model fitting of iron tailings for PAM molecular weight of 300w and four mass concentration (0.01%, 0.04%, 0.08% and 0.16%) of PAM. The measured value represents measured infiltration rate, the analog value P represents the Philip model fitting value of infiltration rate, the analog value h represents the Horton model fitting value of infiltration rate, and the analog value k represents the Kostiakov model fitting value of infiltration rate.

To describe how well each of the models fit more clearly, a fitting effect diagram of the three infiltration models was constructed, and the treatment of 300w molecular weight PAM was selected as an example. Figure 7 shows that the Horton model fitting curve showed rapid decline initially followed by rapid stability. There was a large deviation between the measured infiltration curve and the fitting curve at the corners of infiltration from fast to slow. The change in the S value in the Philip model that characterizes the infiltration capacity did not match with the actual measurements. The whole fitting curve for the Kostiakov model fit the measured values most closely, which best described the entire spectrum of infiltration of iron tailings. Therefore, the Kostiakov model was the best fit model.

### Water retention

#### Cumulative evaporation

**Figure 8:**
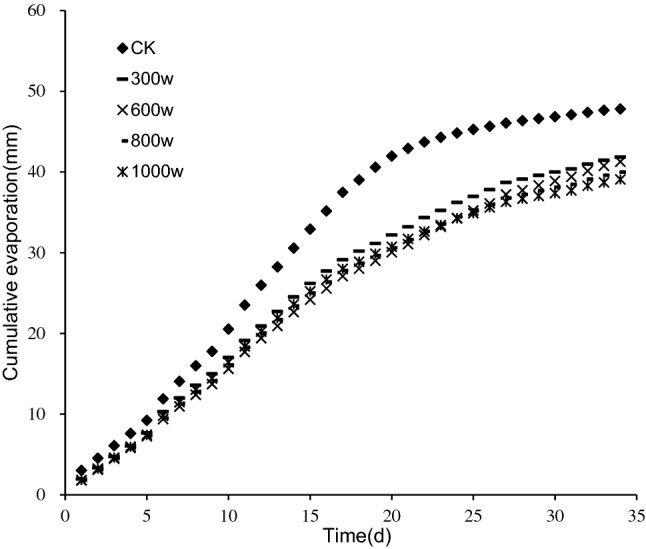
Cumulative evaporation of iron tailings for four molecular weight (300w, 600w, 800w and 1000w) and mass concentration with 0.16% of PAM.

According to our previous research on the relationship between water content and water suction of tailings, when the iron tailings are saturated, the water suction was zero; the water suction of iron tailings increased gradually with the decrease of water content^9^. In the initial stage of evaporation, iron tailings had larger evaporation rate because of smaller water suction, but the water suction of iron tailings increased gradually with the loss of water, the binding force on water got stronger and stronger, and the evaporation rate became smaller and smaller. Therefore, the cumulative evaporation of iron tailings in different treatments showed a trend of steep first and then slow (Fig. 8). However, the rapid evaporation duration of CK and tailings adding PAM was different, the evaporation rate began to decrease after 20 days for CK, and that of tailings adding PAM started to decrease after 30th day. The addition of PAM increased the water suction of tailings and reduced the water evaporation, the cumulative evaporation gradually decreased with the increase of PAM molecular weight. The cumulative evaporation of treatments of PAM with molecular weights of 300w, 600w, 800w, and 1000w on the 35th day was 41.85, 41.27, 39.98, and 39.08 mm, respectively, which decreased by 12.45%, 13.66%, 16.36%, and 18.24% compared with 47.80 mm of CK, respectively. This result indicates that PAM effectively enhanced water retention of tailings. The water retention effect increased with increased PAM molecular weight, but there was no significant difference between PAM molecular weight treatments (*P* < 0.05).

## Discussion

### Effect of saturated hydraulic conductivity and water retention

The effect of PAM on soil saturated hydraulic conductivity is controversial. Some scholars believe that the application of PAM will cause a decrease in soil saturated hydraulic conductivity^23,24^. For this reason, Han et al.^24^ explored the dynamic effect of PAM on saturated hydraulic conductivity of sandy loam and clay, and the results showed that the saturated hydraulic conductivity of different treatments was significantly higher than that of CK at the initial stage of PAM application. However, with the dissolution of PAM, the saturated hydraulic conductivity began to decrease and eventually stabilized at a rate lower than CK. This may be because the molecular chains of PAM are continuously stretched and elongated after hydrolysis, which blocks soil pores in this process. The viscous characteristics of PAM itself may also reduce the saturated hydraulic conductivity. In this experiment, the saturated hydraulic conductivity of the iron tailings under the four molecular weights of 300w, 600w, 800w, and 1000w were all lower than CK during the whole experiment process. However, after a dry–wet cycle, the saturated hydraulic conductivity with molecular weights of 300w, 600w, and 800w (mass concentration 0.16%) were significantly higher compared to before the dry–wet cycle (*P* < 0.05), but still lower than CK. The experiment after a dry–wet cycle was carried out after the soil column underwent evaporation (35 days) and re-saturation. During the process, the loss of PAM or decrease in water absorption capacity may increase the saturated hydraulic conductivity. The effect of PAM on saturated hydraulic conductivity is complicated as it is related to the texture, porosity, aggregate distribution of the soil, and the PAM type. However, the results suggest that the selected PAM cannot increase the saturated hydraulic conductivity of iron tailings, and whether PAM with lower molecular weight or lower concentration could increase the saturated hydraulic conductivity of iron tailings needs further experimentation.

### Effect of soil water infiltration

The effect of PAM on soil water infiltration varied depending on PAM molecular weight and mass concentration. Studies have shown that the application of PAM can increase soil water infiltration^25,26^, but this effect is not absolute. Generally speaking, the higher the molecular weight and mass concentration of PAM, the better the water retention effect. However, if the mass concentration of PAM is too high, it will inhibit the infiltration of soil water. Therefore, when applied, PAM of an appropriate molecular weight should be selected according to the characteristics of soil or tailings. Because the iron tailings are fine and compact, PAM with a smaller molecular weight should be selected^27^. In this study, four kinds of PAM with molecular weights of 300w, 600w, 800w, and 1000w, and five mass concentrations of 0%, 0.01%, 0.04%, 0.08%, and 0.16%, were selected. The research showed that when the molecular weight and mass concentration of PAM was low, PAM had a greater effect on increasing water infiltration and water retention in iron tailings. However, when the molecular weight and mass concentration of PAM increased, the saturated water content and the depth of the water saturated zone of iron tailings increased, but the water infiltration and saturated hydraulic conductivity of tailings was inhibited. The application of PAM alone may alter some of a soil’s limiting factors, but it would be a challenge to meet all needs. To optimize soil improvements, PAM can be mixed with other amendments. The main amendments currently used for the tailing’s reclamation are PAM, biochar, mushroom substrate waste, bentonite, and various other organic wastes^28–30^. Combined with the current research results, mixing PAM with other amendments may achieve better effects^31^, but this needs further research.

## Conclusion

In this research, the different molecular weights and mass concentrations of PAM had different effects on soil water transport. When the PAM with low molecular weight and mass concentrations were added to tailings, it increases the water infiltration and water retention of iron tailings. When PAM with higher molecular weight and mass concentrations were applied to tailings, the saturated water content and depth of the saturation zone of the iron tailings increased, but the PAM also inhibit the water infiltration and saturated hydraulic conductivity of tailings. Therefore, PAM may be most effective in practice if applied by layers in the term of molecular weight and mass concentrations. Applying PAM with lower molecular weight and mass concentration to tailings top layers would be beneficial to soil and water conservation. Applying PAM with higher molecular weight and mass concentration to lower layer tailings could help meet plant root water demands. Future research will focus on the application of PAM layer-by-layer or mixed with other soil amendments to improve the limiting factors of vegetation restoration in tailing areas.

## Materials and methods

### Experimental materials

The iron tailings used for the experiment were taken from an iron tailings pond located at Yuanqu County, Southwestern Shanxi Province in China (34° 59′ N, 111° 30′ E). The bulk density of the tailings at the site was 1.65 g/cm^3^. After the samples were collected and brought back to the laboratory, they were air-dried, crushed, and sieved through a 2 mm soil sieve. Before the experiment, the particle size distribution of iron tailings was measured with a MS2000 laser particle size analyzer^32^, and the iron tailings sample contained 22.12% clay (diameter of < 0.002 mm), 57.29% silt (diameter of 0.002–0.02 mm), and 20.59% fine particles (diameter of 0.02–2 mm). The texture of the iron tailings was classified as sandy clay loam based on the International Classification System of Soil Texture^33^. The organic matter content of the tailings was 2.49 g·kg^−1^ using the potassium dichromate oxidation-external heating method. The anionic polyacrylamides (PAM) used in the experiment with molecular weights of 300w, 600w, 800w, and 1000w, which were produced by Henan Zhongbang Environmental Protection Technology Corporation in China, with a degree of hydrolysis of 30% and a particle size of about 80 mesh.

### Experimental design

The experiment was carried out at the Experimental Station of College of Resources and Environment of Shanxi Agricultural University in China, from May to July 2020. The experiment had a two-factor design. PAM had 4 molecular weights of 300w, 600w, 800w, and 1000w, and 5 mass concentrations of 0% (CK), 0.01%, 0.04%, 0.08%, and 0.16%, for a total of 17 treatments with each treatment in replicates of 3, for a total of 51 groups.

### Experimental measurements

#### Measurement of saturated hydraulic conductivity

**Figure 9:**
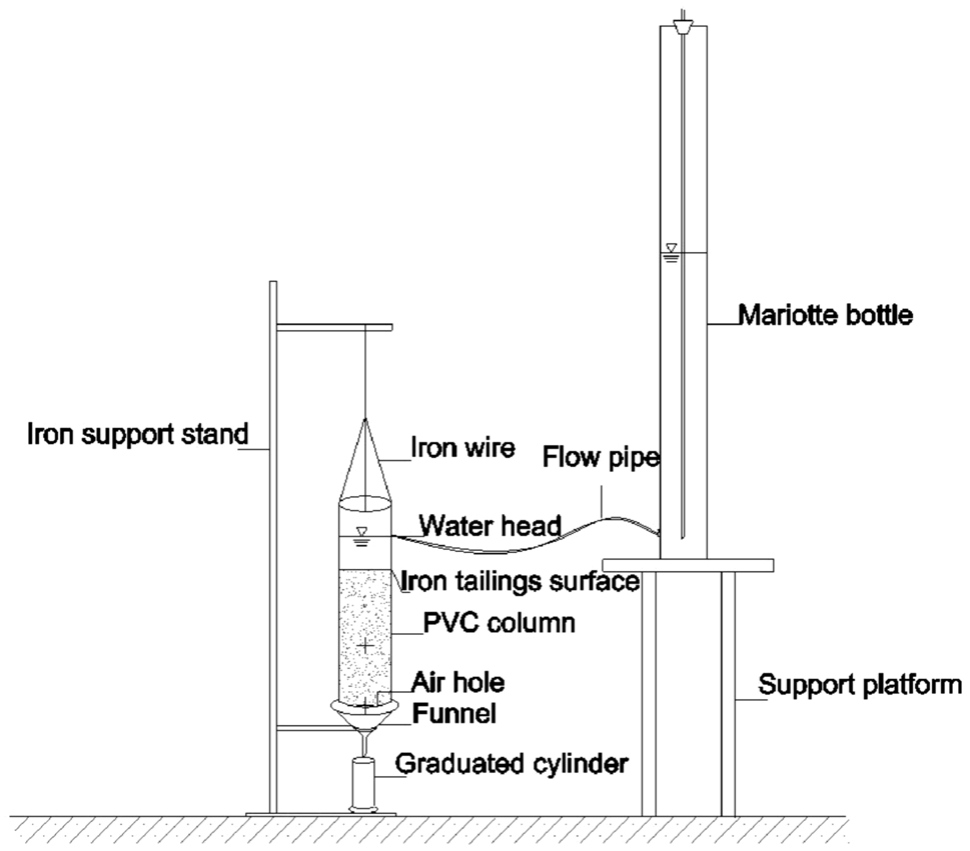
Schematic diagram of experimental setup of saturated hydraulic conductivity.

The saturated hydraulic conductivity was measured using the permeation bucket method. Tailings and PAM were mixed uniformly according to the experimental design and then loaded into transparent polyvinylchloride (PVC) columns. PVC columns had an inner diameter of 8 cm, a height of 30 cm, and the bottom of each PVC column was padded with a filter paper. Each column was filled with iron tailings to a height of 20 cm with a bulk density of 1.65 g/cm^3^, and the columns were filled layer-by-layer at 5 cm intervals. The iron tailings were disturbed between layers to hinder the stratification. Before the experiment began, these PVC soil columns were immersed in water to fully saturate, and the iron tailings surface was covered with filter paper to avoid erosion. A Mariotte bottle was used to supply water to sustain a constant 5 cm-water head (Fig. 9).

Experimental data were recorded once every 5 min when the water flowed through the soil column continuously without bubbles, and the recording stopped after three consecutive stable observations. The saturated hydraulic conductivity was calculated using the following formula^34^:

1 $$ {\text{K}}_{{\text{S}}} = \frac{Q \times L}{{A \times t \times H}} $$

where K_S_ represents saturated hydraulic conductivity (cm min^−1^), Q represents permeation water volume (ml), L represents permeation length (cm), A represents permeation cross-sectional area (cm^2^), t represents permeation time (min), and H represents height of water head (cm).

#### Measurement of water retention

After finishing the saturated hydraulic conductivity measurements, the soil columns of CK and different PAM molecular weights (mass concentration was 0.16%) were kept at a constant indoor temperature environment under natural ventilation.

Water retention in iron tailings is reflective of the daily evaporation capacity. The soil cumulative evaporation can reflect soil water retention capacity. Soil evaporation is mainly affected by soil particle size distribution. If the soil texture is stickier and the soil particles are smaller, the soil capillaries are smaller and the evaporation capacity is larger. Studies have shown that PAM can effectively improve soil water retention; when the PAM molecular weight is constant, increased PAM mass concentration improves soil water retention^16,18^. Therefore, the maximum PAM mass concentration of 0.16% in the experimental design was selected to observe the effect of PAM molecular weight on the water holding capacity of iron tailings.

The evaporation capacity was carried out with soil columns (inner diameter of 8 cm and a height of 30 cm) in a stable indoor environment (the average indoor temperature was 24.3℃). The water loss resulting from soil evaporation was weighed with an electronic scale every afternoon for 36 days until the daily evaporation capacity remained constant. The daily evaporation was calculated as follows^32^:

2 $$ {\text{E}} = {\text{M}}_{{\text{d}}} \times {{10} \mathord{\left/ {\vphantom {{10} {\left( {\uppi {\text{r}}^{2} } \right)}}} \right. \kern-\nulldelimiterspace} {\left( {\uppi {\text{r}}^{2} } \right)}} $$

where E represents daily evaporation of the soil column (mm); M_d_ represents daily change in mass of the soil column (g); and r represents radius of the soil column (cm).

Then the soil columns were saturated again, and the saturated hydraulic conductivity of the iron tailings was measured again to compare with the saturated hydraulic conductivity measured before a dry–wet cycle.

#### Measurement of water infiltration

**Figure 10:**
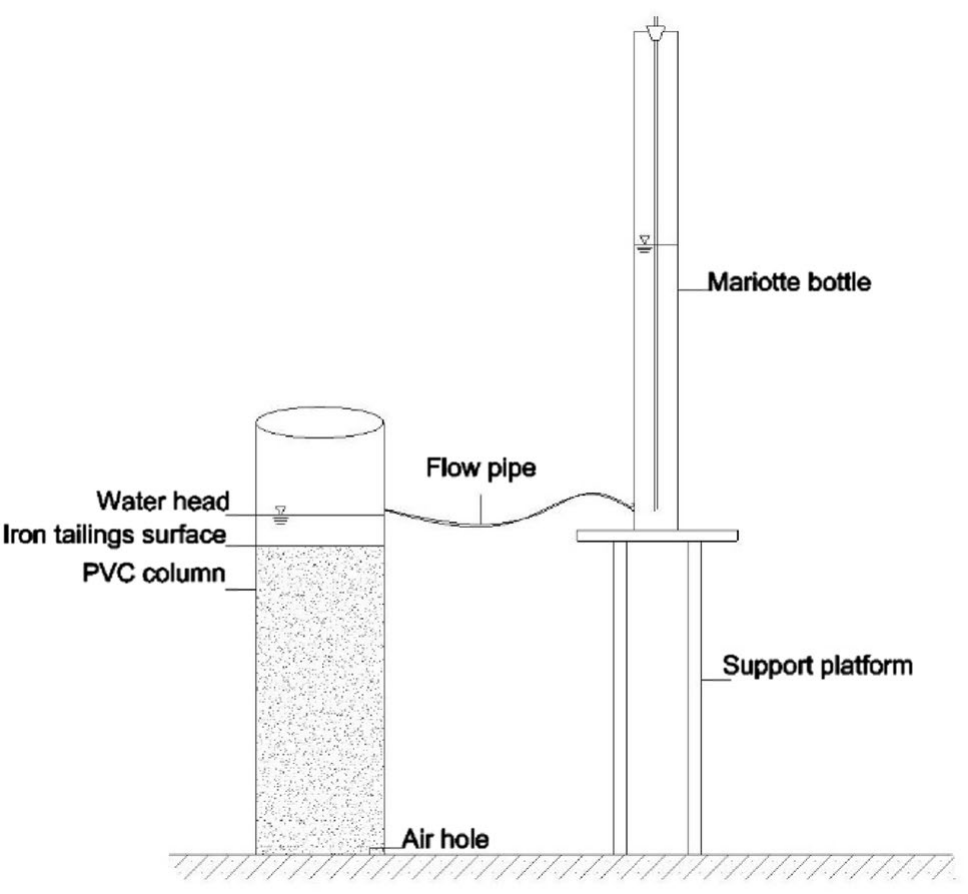
Schematic diagram of experimental setup of water infiltration.

The water infiltration experiment was conducted using a one-dimensional indoor soil column simulation method. Before the experiment, the iron tailings samples were evenly filled into the soil column in layers at 5 cm intervals up to 50 cm (The treatments were the same as the saturated hydraulic conductivity experiment.). The PVC columns had an inner diameter 20 cm and a height of 70 cm. A Mariotte bottle was used to supply water to sustain a constant 5 cm-water head (Fig. 10). The wet peak distance in soil column and the water level in the Mariotte bottle with time were recorded. Once the infiltration rate of tailings stabilized, the water content of different depths of the iron tailings columns were measured using the drying method.

#### Water infiltration model selection

The water infiltration model is a physical model that describes soil infiltration on the basis of a large amount of infiltration data. The infiltration parameters reflect the characteristics of water infiltration under different conditions from a hydrodynamic perspective. In this research, Horton model^35^, Philip model^36^, and Kostiakov model^37^ were used to simulate the vertical movement of water in iron tailings, and the suitability of each model for water infiltration in iron tailings was compared. The expressions of the three infiltration models are:

3 $$ {\text{i}}\left( {\text{t}} \right) = \upalpha {\text{e}}^{{ - {\text{kt}}}} + {\text{c }} $$

4 $$ {\text{i}}\left( {\text{t}} \right) = 0.5{\text{St}}^{ - 0.5} + {\text{A }} $$

5 $$ {\text{i}}\left( {\text{t}} \right) = {\text{at}}^{ - b} $$

where i(t) represents infiltration rate (cm min^−1^); t represents infiltration time (min); S represents water absorption rate (cm min^−1/2^); A and c represent stable infiltration rate (cm min^−1/2^); a, b, and k represent infiltration experience parameters.

### Data analysis

The data measured in the experiment was organize and calculate with Microsoft Excel (version 2019). The statistical analysis was performed with SAS (version 9.4). Analysis of variance (ANOVA) was used to examine differences in the saturated water content, infiltration rate, saturated boundary depth among four PAM molecular weights and five mass concentrations. Significant differences were determined using the Duncan’s new multiple range test with *P* < 0.05. The water infiltration model curve fitting used First Optimization (version 5.0) which was developed by 7D-Soft High Technology Inc. in China.

## References

[1] Tang L, Liu XM, Wang XQ, Liu ST, Deng H (2020). Statistical analysis of tailings ponds in China. J. Geochem. Explor..

[2] Chen R, Zhang L, Budhu M (2013). Biopolymer stabilization of mine tailings for dust control. J. Geotech. Geoenviron. Eng..

[3] Liu BH, Wang SX, Wang J, Zhang XZ, Shen ZG, Shi L, Chen YH (2020). The great potential for phytoremediation of abandoned tailings pond using ectomycorr-hizal *Pinus sylvestris*. Sci. Total Environ..

[4] Lv CJ, Bi RT, Guo XX, Chen D, Guo YS, Xu ZJ (2020). Erosion characteristics of different reclaimed substrates on iron tailings slopes under simulated rainfall. Sci. Rep..

[5] Esposito F, Del Nobile MA, Nicolais L (2015). Water sorption in cellulose-based hydrog-els. J. Appl. Polym. Sci..

[6] Lu SJ, Wang ZL, Hu YX, Liu BY, Liu JE (2018). Effectiveness and durability of polyacrylamide (PAM) and polysaccharide (Jag C 162) in reducing soil erosion under simulated rainfalls. Water.

[7] Song, Y. J., Huang, Y. H., Yang J., Zuo, J. C., Liao, K. T. & Xiao, L. Response of runoff and sediment to PAM in typical soil slopes of South China. *Trans. Chin. Soc. Agric. Mach*. **48,** 279–287 (in Chinese) (2017).

[8] Liang XQ, Liu ZW, Chen LL, Tian GM (2017). Soil colloidal P release potentials under various polyacrylamide addition levels. Land Degrad. Dev..

[9] LÜ, C. J., Wang, Y., Bi, R. T., Liang, J. C., Zhu, H. F. & Chen, D. Comparison of hydraulic characteristics of iron tailings and soil under different compaction levels. *J. Soil Water Conserv*. **34**(01), 108–115+120 (in Chinese) (2020).

[10] Flanagan DC, Norton LD, Shainberg I (1997). Effect of water chemistry and soil amendments on a silt loam soil, Part I. Infiltration and runoff. Trans. ASAE.

[11] Tümsava Z, Kara A (2011). The effect of polyacrylamide (PAM) applications on infiltrateon, runoff and soil losses under simulated rainfall conditions. Afr. J. Biotech..

[12] Bissonnais YL, Singer MJ (1992). Crusting, runoff, and erosion response to soil water content and successive rainfalls. Soil Sci. Soc. Am. J..

[13] Zhang XC, Miller WP (1996). Polyacrylamide effect on infiltration and erosion in fur-rows. Soil Sci. Soc. Am. J..

[14] McLaughlin RA, Bartholomew N (2007). Soil factors influencing suspended sediment F-locculation by polyacrylamide. Soil Sci. Soc. Am. J..

[15] Yu, J., Lei, T. W., Shainberg, I., Zhang, J. S. & Zhang, J. P. Effects of molecular weight and degree of hydrolysis of PAM on infiltration and erosion of y soil. *Acta Pedologica Sinica*. **48**(01), 21–27 (in Chinese) (2011).

[16] Yun, X. F., Wang, Y. K., Wu, P. T., Feng, H. Effects and mechanism of PAM on soil physical characteristics. *J. Soil Water Conserv*. **19**(02), 37–40 (in Chinese) (2005).

[17] Trout TJ, Sojka RE, Lentz RD (1995). Polyacrylamide effect on furrow erosion and infiltration. Dis. Chest.

[18] Fei YH, She DL, Gao L, Xin P (2019). Micro-CT assessment on the soil structure and hydraulic characteristics of saline/sodic soils subjected to short term amendment. Soil Tillage Res..

[19] Pan, Y. H., Lei, T. W., Zhang, Q. W., Liu, G. J. & Xia, W. S. Effects of polyacrylamide on soil hydrodynamic parameters. *Trans. Chin. Soc. Agric. Eng*. **19**(04), 37–39 (in Chinese) (2003).

[20] Sepaskhah AR, Shahabizad V (2010). Effects of water quality and PAM application rate on the control of soil erosion, water infiltration and runoff for different soil textures measured in a rainfall simulator. Biosyst. Eng..

[21] Sojka RE, Bjorneberg DL, Entry JA, Lentz RD, Orts WJ (2007). Polyacrylamide in agriculture and environ mental land management. Adv. Agron..

[22] Li S, Xu H, Ao C (2019). Polyacrylamide and rill flow rate effects on erosion and ammonium nitrogen losses. Water Air Soil Pollut..

[23] Young MH, Moran EA, Yu ZB, Zhu JT, Smith DM (2009). Reducing saturated hydraulic conductivity of sandy soils with polyacrylamide. Soil Sci. Soc. Am. J..

[24] Han, D., Wei, Z. M., Yu, J. & Song, R. Q. Dynamic effect of the dissolution time of PAM on soil saturated hydraulic conductivity. *Soils*. **48**(02), 368–373 (in Chinese) (2016).

[25] Santos FL, Serralheiro RP (2000). Improving infiltration of irrigated mediterranean soilswith polyacrylamide. J. Agric. Eng. Res..

[26] Ajwa H (2011). Polyacrylamide effect on infiltration of various quality water. Hybridoma.

[27] Green VS, Stott DE, Norton LD, Graveel JG (2000). Polyacrylamide molecular weight and charge effects on infiltration under simulated rainfall. Soil Sci. Soc. Am. J..

[28] Li, X., Zhang, B. J., Li, J. Q., Li, Y. L. & Li, C. G. Effects of combined application of water retention agent and organic fertilizer on physic-chemical properties of iron tailings. *Chin. J. Appl. Ecol*. **28,** 554–562 (in Chinese) (2017). 10.13287/j.1001-9332.201702.036 29749164

[29] Acosta JA, Abbaspour A, Martinez GR, Martinez-Martinez S, Zornoza R, Gabarron M, Faz A (2018). Phytoremediation of mine tailings with *Atriplex halimus* and organic/inorganic amendments: a five-year field case study. Chemosphere.

[30] Benidire L, Madline A, Pereira SIA, Castro PML (2020). Synergistic effect of organomineral amendments and plant growth promoting rhizobacteria (PGPR) on the establishment of vegetation cover and amelioration of mine tailings. Chemosphere.

[31] Lee SS, Shah HS, Awad YM, Kumar S, Ok YS (2015). Synergy effects of biochar and polyacrylamide on plants growth and soil erosion control. Environ. Earth Sci..

[32] Konert M, Vandenberghe J (2010). Comparison of laser grain size analysis with pipette and sieve analysis: a solution for the underestimation of the clay fraction. Sedimentology.

[33] Atterberg A (1912). Die mechanische bodenanalyse und die klassifikation der mineralböden schwedens. Internationale Mitteilungen für Bodenkunde..

[34] Cheng, D. J., Zhang, Y. L., et al. *Soil Physics Experiment Guide; Scientific*. Published by Water & Power Press, China (in Chinese) (2012).

[35] Horton RE (1940). An approach toward a physical interpretation of infiltration capacity. Soil Sci. Soc. AM. Proc..

[36] Philip JR (1957). The theory of infiltration about sorptivity and algebraic infiltration equations. Soil Sci..

[37] Kostiakov, A. N. On the dynamics of the coefficient of water-percolation in soils and on the necessity of studying it from a dynamic point of view for purposes of amelioration. In *Trans. 6th Comm. Int. Soc. Soil. Russian*, Vol. 97, 17–21 (1932).

